# Cotton Fusarium wilt diagnosis based on generative adversarial networks in small samples

**DOI:** 10.3389/fpls.2023.1290774

**Published:** 2023-12-11

**Authors:** Zhenghang Zhang, Lulu Ma, Chunyue Wei, Mi Yang, Shizhe Qin, Xin Lv, Ze Zhang

**Affiliations:** ^1^ Xinjiang Production and Construction Crops Oasis Eco-Agriculture Key Laboratory, Shihezi University College of Agriculture, Shihezi, China; ^2^ Natiobal-Local Joint Engineering Research Center of Xinjiang Production and Construction Corps XPCC's Agricultural Big Data, Shihezi, China; ^3^ Agricultural Development Service Center, Fifty-first Mission, Third Division, Tumushuke, China

**Keywords:** cotton diseases, data augmentation, transfer learning, generative adversarial networks, convolutional networks

## Abstract

This study aimed to explore the feasibility of applying Generative Adversarial Networks (GANs) for the diagnosis of Verticillium wilt disease in cotton and compared it with traditional data augmentation methods and transfer learning. By designing a model based on small-sample learning, we proposed an innovative cotton Verticillium wilt disease diagnosis system. The system uses Convolutional Neural Networks (CNNs) as feature extractors and applies trained GAN models for sample augmentation to improve classification accuracy. This study collected and processed a dataset of cotton Verticillium wilt disease images, including samples from normal and infected plants. Data augmentation techniques were used to expand the dataset and train the CNNs. Transfer learning using InceptionV3 was applied to train the CNNs on the dataset. The dataset was augmented using GAN algorithms and used to train CNNs. The performances of the data augmentation, transfer learning, and GANs were compared and analyzed. The results have demonstrated that augmenting the cotton Verticillium wilt disease image dataset using GAN algorithms enhanced the diagnostic accuracy and recall rate of the CNNs. Compared to traditional data augmentation methods, GANs exhibit better performance and generated more representative and diverse samples. Unlike transfer learning, GANs ensured an adequate sample size. By visualizing the images generated, GANs were found to generate realistic cotton images of Verticillium wilt disease, highlighting their potential applications in agricultural disease diagnosis. This study has demonstrated the potential of GANs in the diagnosis of cotton Verticillium wilt disease diagnosis, offering an effective approach for agricultural disease detection and providing insights into disease detection in other crops.

## 1 Introduction

A prominent disease that affects cotton production, Fusarium wilt poses considerable challenges to the growth and development of cotton. This destructive disease can spread through various means, including infected cotton seeds, plant residues, soil, water sources, and farming tools. Once infected, the pathogen rapidly reproduces within the plant, triggering an immune response resulting in leaf yellowing, flower bud shedding, and reduced yield. In severe cases, the entire plant may experience extensive death, leading to catastrophic consequences such as complete crop failure. In normal years, economic losses of 5–15% are caused by this disease. In certain years, owing to timely prevention and incorrect interventions, economic losses can reach 30–50% (Li et al., 2011).

Therefore, ensuring healthy development of the cotton industry necessitates the detection and control of Fusarium wilt. Traditional detection methods rely heavily on manual observation and pathogen identification, and are time-consuming, labor-intensive, and susceptible to subjective factors. To enhance the accuracy and efficiency of detection, there has been increasing research interest in using Convolutional Neural Networks (CNN) for automated Fusarium wilt detection (Goodfellow et al., 2014). By training the CNN model with a large number of Fusarium wilt samples, the model can learn disease characteristics and accurately classify and identify diseases. Traditional manual identification of Fusarium wilt not only lacks precision, but also incurs high time and labor costs. The accurate identification of Fusarium wilt is crucial in the cotton industry. Implementing intelligent identification methods is essential for the effective differentiation and diagnosis of this destructive disease.

However, given the difficulty in obtaining Fusarium wilt samples, improving the robustness and generalization ability of the model is severely limited. Therefore, exploring new methods to expand sample sizes is of great significance for the detection and control of Fusarium wilt in cotton, thereby benefiting the economic growth of China’s cotton industry. With the development of deep learning technology, it has been widely applied in crop analysis. In the monitoring of Fusarium wilt in cotton, the application focus has mainly been on transfer learning and hyperspectral imaging or training classification networks to recognize and differentiate diseases and pests. Classical learning often requires large amounts of training data. Training a qualified network without sufficient support in terms of sample quantity or quality is challenging.

To address the problem of limited experimental data collection, Generative Adversarial Networks (GANs) are used to learn from a small set of fundamental samples to generate realistic images that can be used for the training of discriminative networks. Therefore, this study used a CNN with a sample set generated by GANs for the recognition of Fusarium wilt in cotton and compared it with traditional data augmentation methods and transfer learning approaches. The research methodology presented in this study serves as a reference for the recognition of cotton Fusarium wilt under small-sample conditions to accurately detect and identify the disease.

## 2 Materials and methods

### 2.1 Experimental materials and design

This study focused on cotton wilt disease as the primary research subject. Given the inconvenience of obtaining data and images in the field of agronomy, relevant images of healthy and diseased cotton leaves were obtained from the College of Agriculture at Shihezi University and compiled into a small cotton wilt disease dataset through personal organization and classification.

The dataset consisted of a training set and a validation set. The training set comprised 154 images, including 71 images of healthy cotton leaves and 83 images of diseased cotton leaves. The validation set comprised 78 images, including 36 images of healthy cotton leaves and 42 images of diseased cotton leaves. The test set included 185 images, with 118 of healthy cotton leaves and 67 of diseased cotton leaves. The original resolution of each image was 569 × 569 pixels; however, after compression, the resolution was 256 × 256 pixels.

### 2.2 Data processing

According to the research network model, targeted adjustments were made based on existing datasets. The original CNN network, the original dataset will be used. For a CNN that uses data augmentation techniques, the sample size is increased through data augmentation. The original dataset was used for the CNN, which uses transfer learning techniques. The dataset generated through the GAN was used for the CNN that incorporated the GAN.

### 2.3 Data augmentation techniques

**Figure 1:**
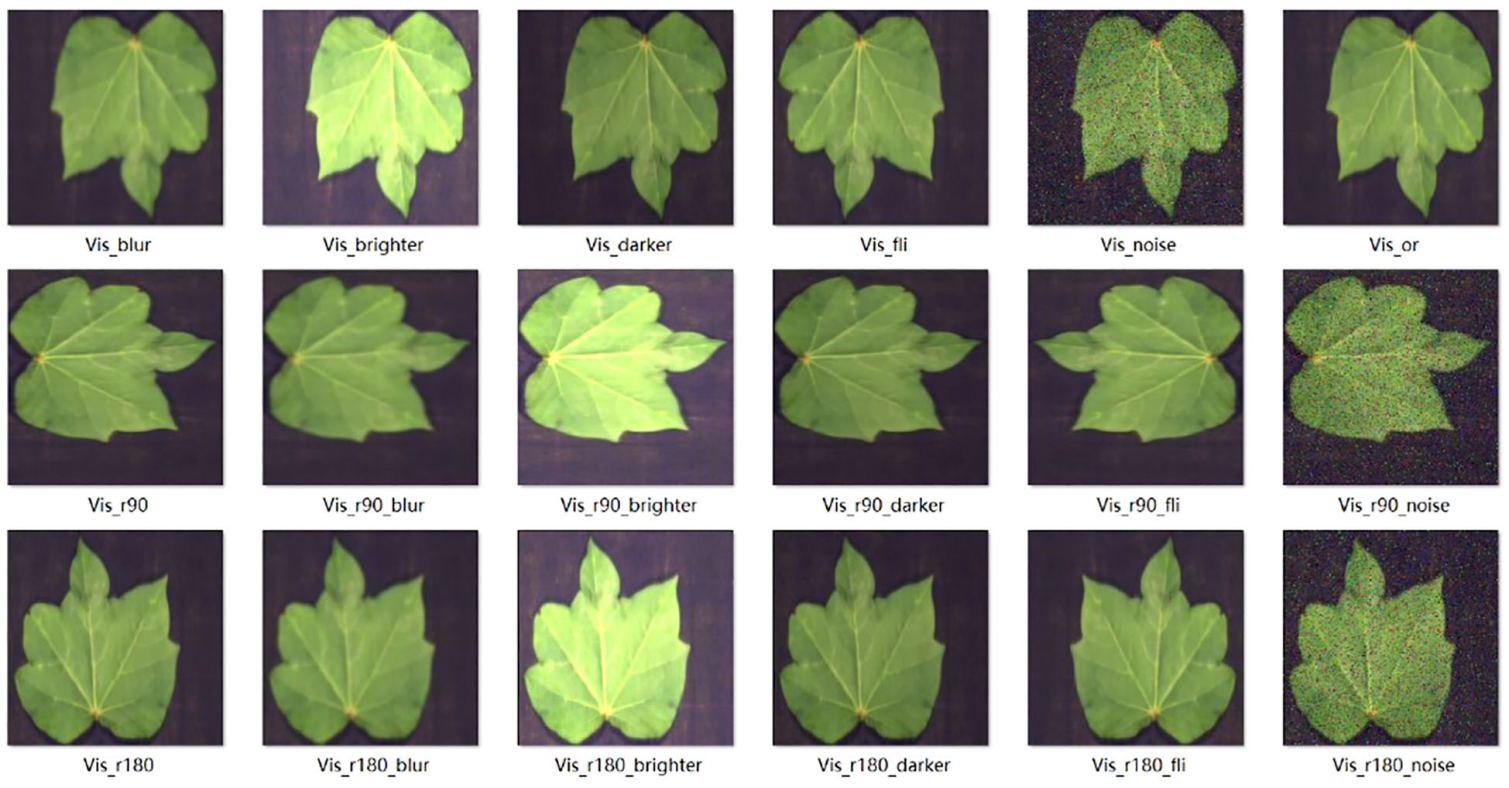
Samples after data augmentation.

The images were enhanced using the following techniques. Salt-and-pepper noise and Gaussian noise were introduced to disrupt the image quality and they were manipulated by rotation and flipping to alter the image orientation. The brightness was adjusted to make the image brighter or darker. Either salt-and-pepper noise or Gaussian noise was applied first, and the brightness was then adjusted accordingly. With these four image enhancement techniques in place, we proceeded with the following rotation operations: clockwise rotation by 90°(r90), counterclockwise rotation by 90°(r90_fil), 180-degree flip (r180), and horizontal flip (fil). After applying noise (noise), the image was darkened (darker), brightened (brighter), blurred (blurred), and the flipping operations were repeated. By implementing this image enhancement process, one original image could yield 16 augmented images, thereby expanding the sample size by a factor of 17, as shown in **Figure 1**.

### 2.4 Transfer learning techniques

The InceptionV3 model was trained on the ImageNet dataset, which encompasses a wide range of categories, such as animals, objects, and scenes. The objective of this training process was to enable the InceptionV3 model to learn the general features of images, thereby facilitating diverse image classification and recognition tasks. Although the training of InceptionV3 does not directly involve the plant pest dataset, researchers have used transfer learning methods to apply them to the plant pest dataset. This can enhance the performance of the model for this specific task through fine-tuning and training. This transfer learning approach leverages the feature representation capabilities of the InceptionV3 model which has learned from extensive image classification tasks to aid plant pest recognition tasks.

In this section, the InceptionV3 network was transferred to the original dataset, and the pretrained layers were frozen, preserving their weights throughout the training process. The pre-trained bottom layers of the InceptionV3 network were frozen, whereas only the newly added fully connected layers were trained and weight-adjusted. This enables training on a small sample dataset while using the feature representation capabilities of the InceptionV3 network acquired from large-scale datasets (Mohanty et al., 2021).

### 2.5 Generative adversarial network techniques

A GAN is a generative model with a unique training mechanism comprising a combination of a generator and discriminator. During the training process, the generator and discriminator were trained alternately. In the training process of the GAN, random points were first sampled from the latent space and then decoded into fake images generated by the generator. These fake images were merged with real samples and labeled with random noise to differentiate between the real and fake images. This labeling process improved the robustness and generalizability of the model.

Next, random points were sampled again from the latent space and merged with the labels. However, at this point, all the labels should be “real images” to deceive the discriminator. At this stage, the weights of the discriminator are frozen, and they do not participate in the training of the model; only the generator is trained. In the generator, the Graph Neural Network (GNN) does not impose heavy requirements on intermediate neural networks, which can be fully connected networks (FC), convolutional networks (CNN), or recurrent networks (RNN). Different networks have different degrees of impact on the quality of generated images. A GNN relies on these networks to generate images.

The types of discriminator networks in a GNN are not limited. The discriminator can be implemented using fully connected networks (FC), convolutional networks (CNN), or recurrent networks (RNN). This flexibility allows the GNN to adapt to various types of data and tasks.

**Figure 2:**
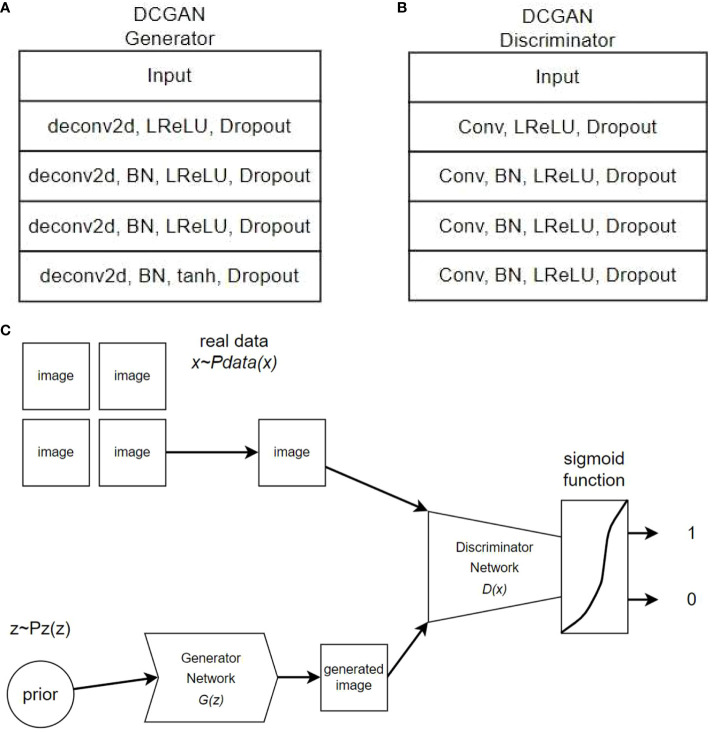
DCGAN Generator、Discriminator and training process.

The architecture of the generator network for the DCGAN and the architecture of the DCGAN used in this study are illustrated in **Figure 2**.

The generator was trained using a GAN model to make it increasingly difficult for the discriminator to distinguish between the generated fake and real images. Through iterative training, the generator produces more realistic images, whereas the discriminator continuously improves its ability to differentiate between real and fake images. The generator and discriminator engage in adversarial training driven by the adversarial loss function, as illustrated in **Figure 2C** of the training process.

### 2.6 Testing with a convolutional network

To ensure test uniformity, we used the same convolutional network for testing and did not include any pre-trained networks. The testing convolutional network comprises five convolutional layers, wherein the parameters double from 32 to 512. Two fully connected layers process and predict the parameters post-convolutional layers. The first fully connected layer features 256 parameters and uses L2 regularization and Dropout techniques for overfitting prevention. The second layer classifies the results employing a sigmoid function. Technical abbreviation explanations are provided when first used.

## 3 Results and analysis

### 3.1 Analysis of training convolutional neural networks on raw data

The hyperparameters of the convolutional neural network were determined experimentally. The specific hyperparameters are listed in **Supplementary Materials**.

**Figure 3:**
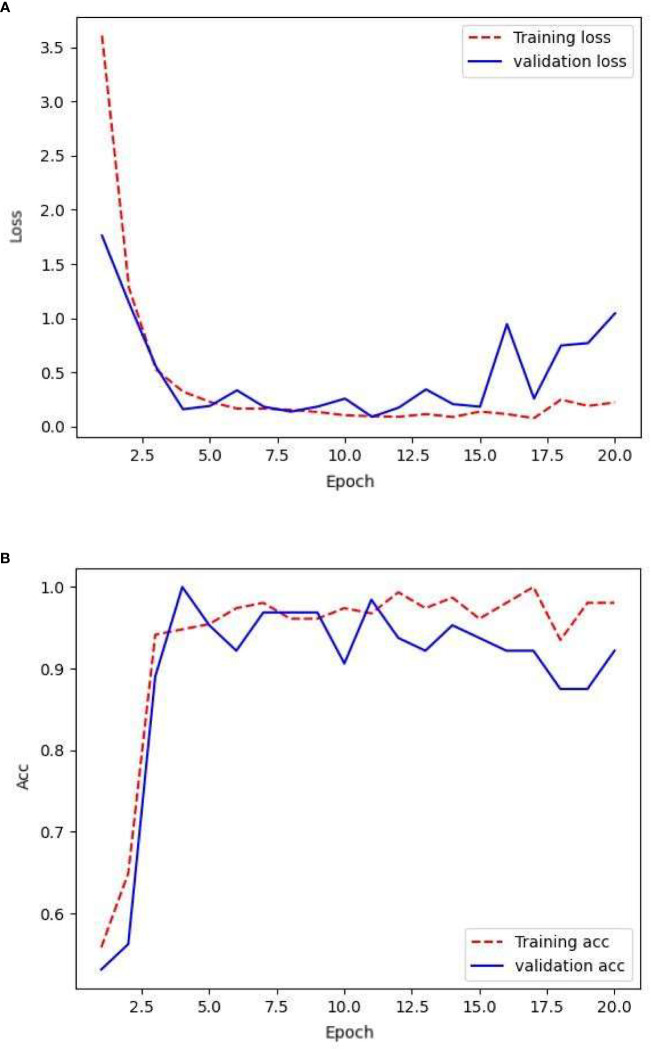
Loss and accuracy under original samples.

The network was trained using the following raw data: 154, 78, and 187 images in the training, validation, and testing sets, respectively. After training the network with the training set, its generalization capability was tested using a validation-set validation. The resulting loss rate and the accuracy are shown in **Figure 3**.

From the given text, it can be observed that the model performed effectively on the training set. The smooth training set loss curve indicates that the CNN could effectively learn from the training set images. However, there were shortcomings in its performance in the validation set. However, the fluctuating nature of the validation set loss curve suggests that the network struggled to recognize the finer details present in the validation set images. Combining this with the accuracy of the validation set, it is clear that the model suffered from severe overfitting. This could be attributed to the limited amount of available data, which prevented the CNN from learning an adequate amount of detail. The overall accuracy of the validation set was lower than that of the training set, but remained at approximately 90%.

**Table 1** compares the accuracy and loss values of the CNN across various datasets at the 20th epoch. By the 20th epoch, the network parameters underwent significant updates. The network trained on the original sample data achieved an accuracy of 98% on the training set but experienced a considerable drop in accuracy on the validation set, reaching 92%. In the independent test set, the level of accuracy decreased to 87%. The loss value of the validation set indicates that the network lacks robustness and stability, potentially because of the limited number of image details provided by the sample data.

**Table 1:** Performance of the network on various datasets with original samples.

Classification	Train	Validation	Test
Precision	0.9805	0.9219	0.8757
Loss Value	0.2223	1.0443	0.3634

### 3.2 Analysis of convolutional network training under data augmentation techniques

**Figure 4:**
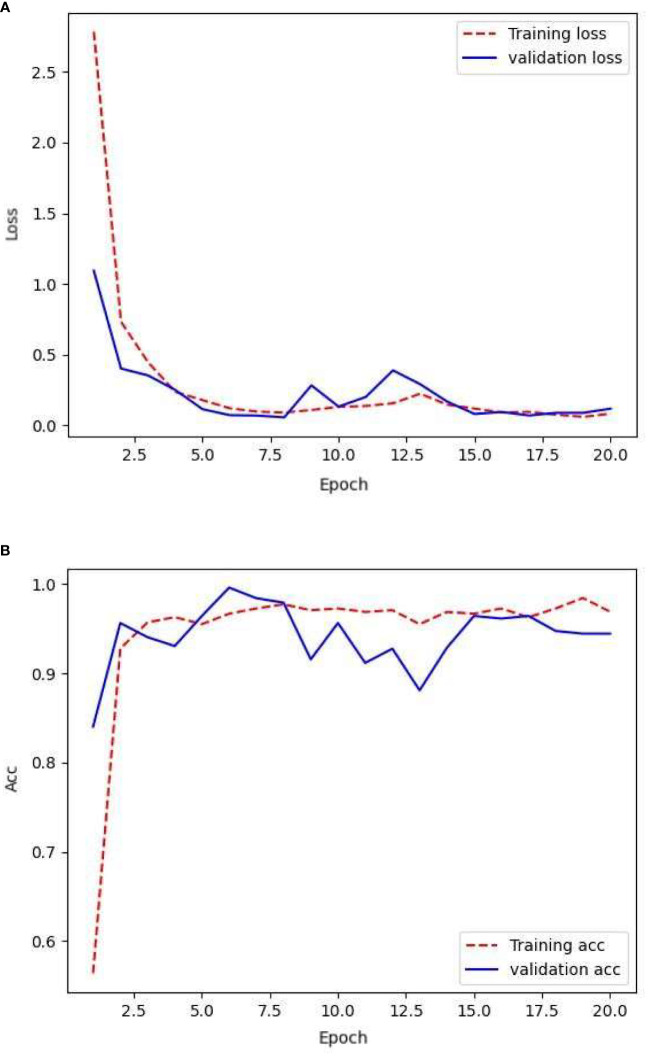
Loss and accuracy under data augumentation.

The augmented dataset consisted of 3204 images for the training set, 1008 images for the validation set, and 187 images for testing the network training. After training the network using the training set, its generalization ability was evaluated using the validation set. The loss rate obtained and its accuracy are shown in **Figure 4**.

These results indicate that throughout the training process, while the training loss approached convergence, the validation accuracy consistently maintained a high level and approached convergence. Only slight overfitting occurred during the training period. In the early stages of network training, the validation loss also approached convergence, showing an overall downward trend. Meanwhile, the validation accuracy approached convergence and gradually improved. There were significant fluctuations in the validation loss and accuracy in the early stages of training, suggesting that the model may not have been sufficiently learned. In the later stages of training, the validation loss approached convergence and exhibited reduced divergence, indicating that the network became more stable. The validation accuracy improved, suggesting that the network reached a relatively stable state after approximately 20 training iterations.

**Table 2** presents a comparison of the accuracy and loss values of the CNN across different datasets at the 20th epoch. With an increased number of training samples, the accuracy improved compared to the original dataset, whereas the loss decreased and approached convergence. Despite a slight decrease in validation accuracy, with an equal proportional increase in the validation set, there was a significant decrease in validation loss, suggesting an improvement in the early stage stability of the network. The model achieved an accuracy of 92% on the test set. However, the model still exhibited overfitting, particularly in the latter half of network training. Here, the validation loss showed divergence and the accuracy experienced significant fluctuations. This can be attributed to the negative impact of repetitive features resulting from data augmentation.

**Table 2:** The performance of the network under data augmentation across different sample sets.

Classification	Train	Validation	Test
Precision	0.9997	.9531	0.9219
Loss Value	0.0112	0.1580	0.2945

### 3.3 Analysis of convolutional network training under transfer learning techniques

After continuous training, the hyperparameters for InceptionV3 have were determined as listed in **Supplementary Materials**.

**Figure 5:**
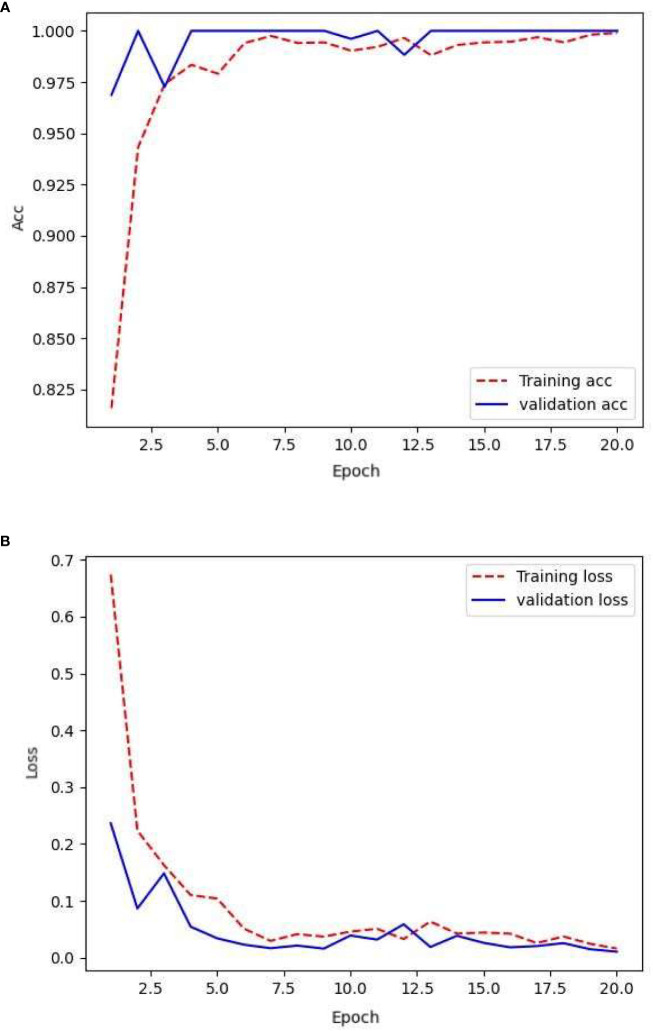
Loss and accuracy under transfer learning.

The network was trained using the initial data, that is, 154 images in the training set, 78 images in the validation set, and 187 images in the test set. After training on the training set, the network generalizability was tested by validating the validation set. The loss rate obtained and its accuracy are shown in **Figure 5**.

From the aforementioned training process, it is evident that the training loss and accuracy increased with each epoch, demonstrating high-quality network training. However, the validation loss and accuracy exhibited significant fluctuations. This has indicated the presence of overfitting in the network and the need for strong regularization techniques. The validation accuracy remains relatively low at approximately 87%. Meanwhile, the validation loss fluctuates within the range of 0.2 to 0.4, suggesting suboptimal generalization capabilities of the model.

**Table 3** presents a comparison of the accuracy and loss values for the CNN across the different datasets after the 20th epoch. Relying on a large-scale dataset for training, along with the complexity of the InceptionV3 network architecture, leads to improved accuracy compared with the original dataset. Based on training with the ImageNet dataset, transfer learning offers more pronounced advantages in terms of parameters compared with a simple CNN network structure (Patil et al., 2019), resulting in a test accuracy of 94%. However, this model also suffers from overfitting, particularly during the validation process, as shown by the fluctuating loss values and accuracy. This highlights the limitation of the ImageNet dataset, which lacked samples related to plant diseases and pests. The significant discrepancy between the original and target domains impedes precise matching, thereby hindering the correct learning of disease-specific details in small-scale datasets.

**Table 3:** Performance of the network with transfer learning on various datasets.

Classification	Train	Validation	Test
Precision	0.9855	0.9375	0.9459
Loss Value	0.0353	0.2557	0.1168

### 3.4 Analysis of convolutional network training under generative adversarial network techniques

After repeated attempts and detailed considerations, an appropriate set of hyperparameters was selected to train the generator model. The learning rate was set to 0.0004, which is a commonly selected value that strikes a balance between overfitting and underfitting. The batch size was set to 16, which enhanced training speed and improved the adaptability of the model to small datasets. We chose 1000 training epochs to ensure that the model thoroughly learned the distinctive features of the training data and achieved optimal training results. We incorporated an L2 regularization term to mitigate overfitting concerns.

To maintain the gradient smoothness and stability, we used the LeakyReLU activation function, setting the output of its negative range to a small fraction of the input. To further stabilize the training process, we implemented the gradient clipping (ClipValue) technique by constraining the gradient values to within 1.0. We used the Adam optimization algorithm with a low decay rate to improve the generalization performance of the model. These hyperparameter selections enabled the model to attain favorable results during the training process, thereby enhancing precision and robustness. A summary of the generator hyperparameters is presented in **Supplementary Materials**.

A learning rate of 0.0001, batch size of 16, and 1000 epochs were selected for the training of the discriminator. To enhance the robustness of the model and prevent overfitting, the LeakyReLU activation function with a parameter of 0.2 and dropout regularization with a parameter of 0.5 were implemented. The decay rate was set to 1e-8 to control the training speed of the model, and the Adam optimization algorithm was used to update the network parameters. These parameter choices were obtained through multiple experiments and fine-tuning to maximize the accuracy and generalization capability of the model. The specific parameters of the discriminator are listed in **Supplementary Materials**.

**Figure 6:**
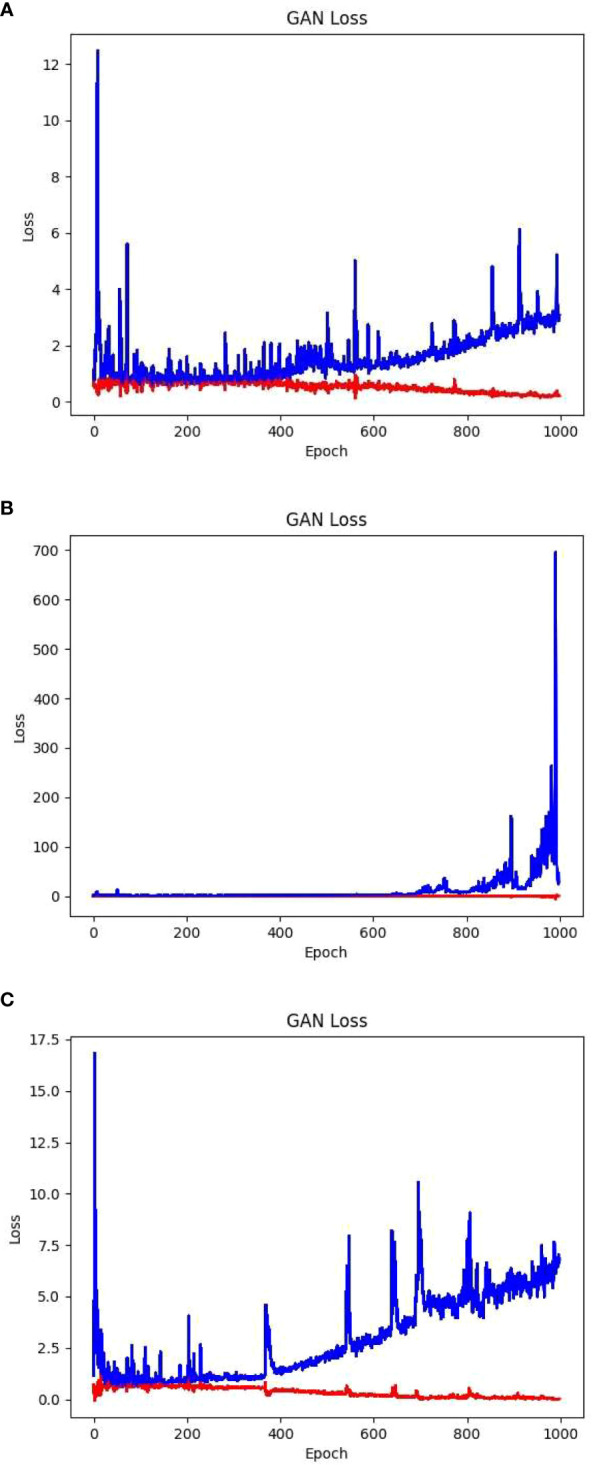
Three different structures of GAN network loss value.

Building on a plethora of examples showing face images generated through GAN, this experiment initially constructed a network structure consisting of convolution–inverse convolution–convolution. The convolutional part begins by applying two layers of convolutional kernels to the input latent vector and extracting the intricate details of the features of the latent vector. Conducting convolution as the initial step effectively reduces the network parameters and computational workload, thus accelerating subsequent inverse convolution operations. Following the inverse convolution layer, a convolution layer is added to extract the features of the generated image and feed them into the discriminator for discrimination. Using the aforementioned hyperparameters and training methodology, the model was trained for 1000 epochs. The training losses (blue for the generator loss and red for the discriminator loss) are illustrated in **Figure 6A**.

**Figure 7:**
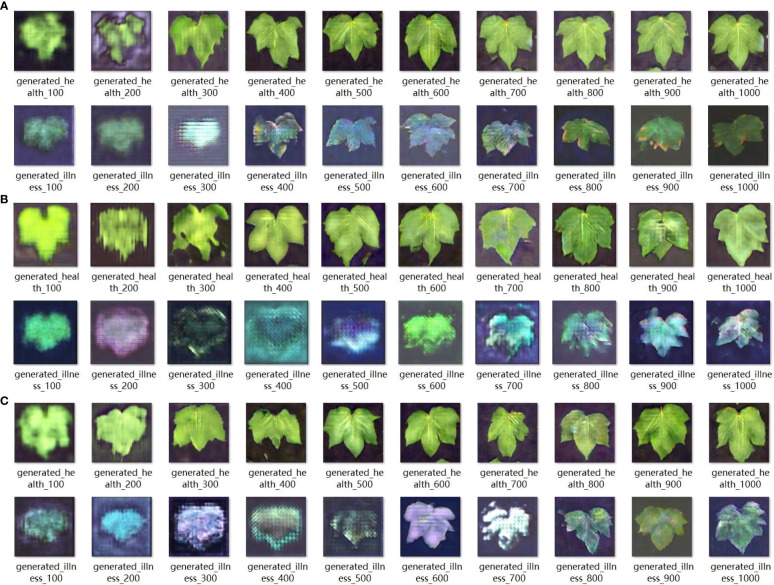
Three different structures of GAN networks generate images every hundred rounds.

The aforementioned loss values showed that as the number of training iterations increased, the generator loss of the GAN gradually increased and reached an unstable state. In the later stages, a gradient explosion occurred. The images generated were of subpar quality with a certain degree of blurriness. Examples of the partially generated images are shown in **Figure 7A**.

The issue reflected in the convolution–deconvolution–convolution model primarily manifested as the loss of certain image features owing to excessive convolution. To address this problem, a convolution–deconvolution structure was used to reduce feature map compression. Although this approach increased the training time and computational complexity, it significantly improved the image quality. Using these hyperparameters and training approach, the training loss, with blue denoting the generator loss and red denoting the discriminator loss, after 1000 training iterations is shown in **Figure 6B**.

The aforementioned loss values showed that as the number of training iterations increased, the generator loss of the GAN gradually increased and remained in an unstable state, whereas the discriminator loss continued to decrease. However, this model overcomes the issue of gradient explosion, allowing the generator loss to remain within an acceptable range. In the later stages, the discriminator loss reached values below 0.0, indicating that the discriminator started dominating the generator generation process. In this scenario, the generator could not generate sufficiently high-quality images. Although it performed well in generating images of healthy leaves, it exhibited shortcomings in generating images of diseased leaf samples, such as inadequate detail and blurry edges. A subset of the generated images is presented in **Figure 7B**.

Considering issues such as the loss of image detail caused by the convolution–deconvolution structure and recognizing that the nature of the foliage is relatively simple, the convolution structure before the deconvolution process was eliminated. Instead, only the basic deconvolution method was used for image restoration. Although this structure increased the computational complexity and training time, it yielded higher image quality and finer edge details. Using the aforementioned hyperparameters and training approach, 1000 training iterations were performed, and the corresponding training losses, with blue representing the generator loss and red representing the discriminator loss, are displayed in **Figure 6C**.

The given loss values have shown that the network loss of this structure is significantly lower than that of the convolution inverse convolution structure, and converges more effectively. During the initial training phase of the network, the generator and discriminator formed a strong adversarial relationship. This enabled the generator to produce images with superior edge details. In the later stages of training, the discriminator began to dominate the generator. In such cases, there is no further need for continued training, as this would exacerbate overfitting in the network. The partially generated images are shown in **Figure 7C**.

After comparing the performances of the three network architectures, the third architecture, specifically the deconvolutional network structure, was chosen to generate a new training set. This dataset was completely generated by a well-trained GAN and consisted of a training set and a validation set. The training set comprised 1600 images of healthy cotton leaves and 1600 images of diseased cotton leaves, for a total of 3200 images. The test set included 400 images of healthy cotton leaves and 400 images of diseased cotton leaves, totaling 800 images. The original resolution of each image is 256 × 256 pixels. Partial representations of the samples are shown in **Supplementary Materials**.

**Figure 8:**
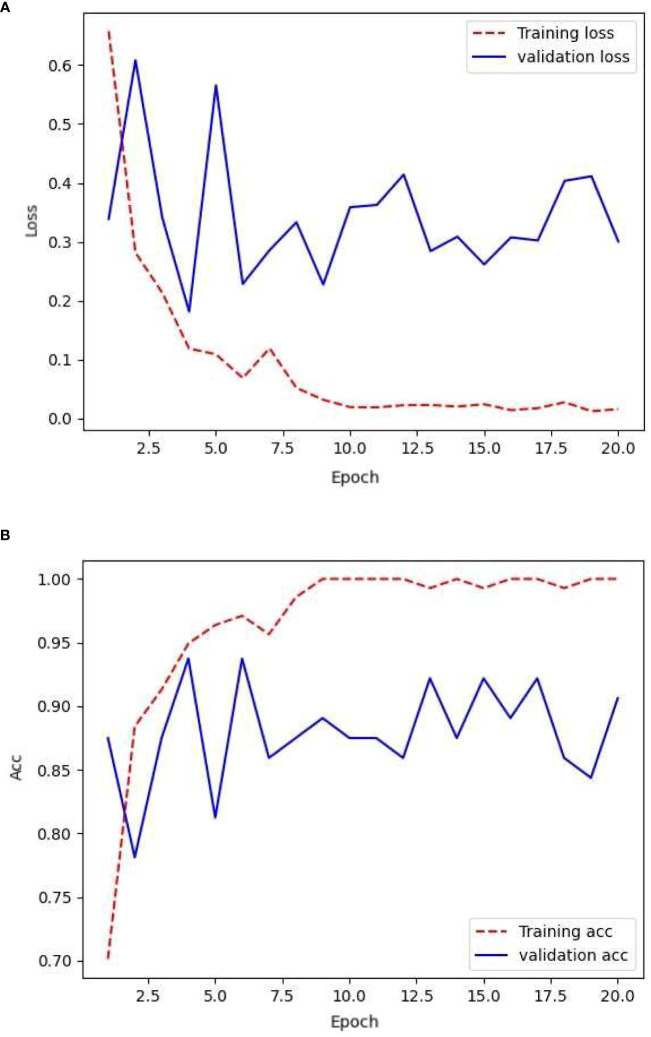
Loss and accuracy during GAN network training with augmented samples.

The network was trained by using the data generated from the GAN network, that is, 3200 images for the training set, 800 images for the validation set, and 187 images for the testing set. After training the network using the training set, its generalization ability was tested using the validation set. The loss rate obtained and its accuracy are illustrated in **Figure 8**.

The model consistently exhibited favorable convergence during the training process. However, a linear fit of the validation accuracy showed a potential overfitting state, where the network may have excessively learned repetitive features from certain samples. However, the model demonstrated high validation accuracy and low validation loss, which were not observed during the training of the first two sample sets. During the training phase, the validation loss remained relatively stable and comparable to the training loss. Overall, the validation accuracy remained consistently higher than the training accuracy, reaching approximately 99%.

**Table 4** provides a comparison of the highest accuracies achieved on the training and validation sets, as well as the accuracy of the test set. The training and validation sets achieved commendable accuracy, surpassing 98% with relatively low loss values. However, the performance of the model on the test set was slightly inferior to that of the training process, with an accuracy of 95%. The model exhibited signs of overfitting during training, potentially owing to interference from a large number of visually similar image pairs in the samples, making it more prone to overfitting. This is a challenge encountered when using GAN.

**Table 4:** Performance of GAN network on different sample sets with augmented samples.

Classification	Train	Validation	Test
Precision	0.9988	0.9922	0.9499
Loss Value	0.0249	0.0249	0.3344

A comparison of the accuracy and loss values for the enhanced dataset using the GAN, augmented dataset, original dataset, and transfer-learning techniques is presented in **Table 5**.

**Table 5:** Comparison between GAN network with augmented samples and transfer learning, data augmentation, and original data.

Classification	Train	Validation	Test
Precision (GAN-Enhanced)	0.9988	0.9922	0.9531
Accuracy (Transfer Learning)	0.9855	0.9375	0.9459
Accuracy (Data Augmentation)	0.9838	0.9286	0.9333
Accuracy (Original)	0.9805	0.9531	0.8973
Loss value (GAN-Enhanced)	0.0249	0.0249	0.3167
Loss value (Transfer Learning)	0.0353	0.2557	0.1168
Loss value (Data Augmentation)	0.0469	0.1095	0.1823
Loss value (Original)	0.1070	0.1470	0.2170

**Figure 9:**
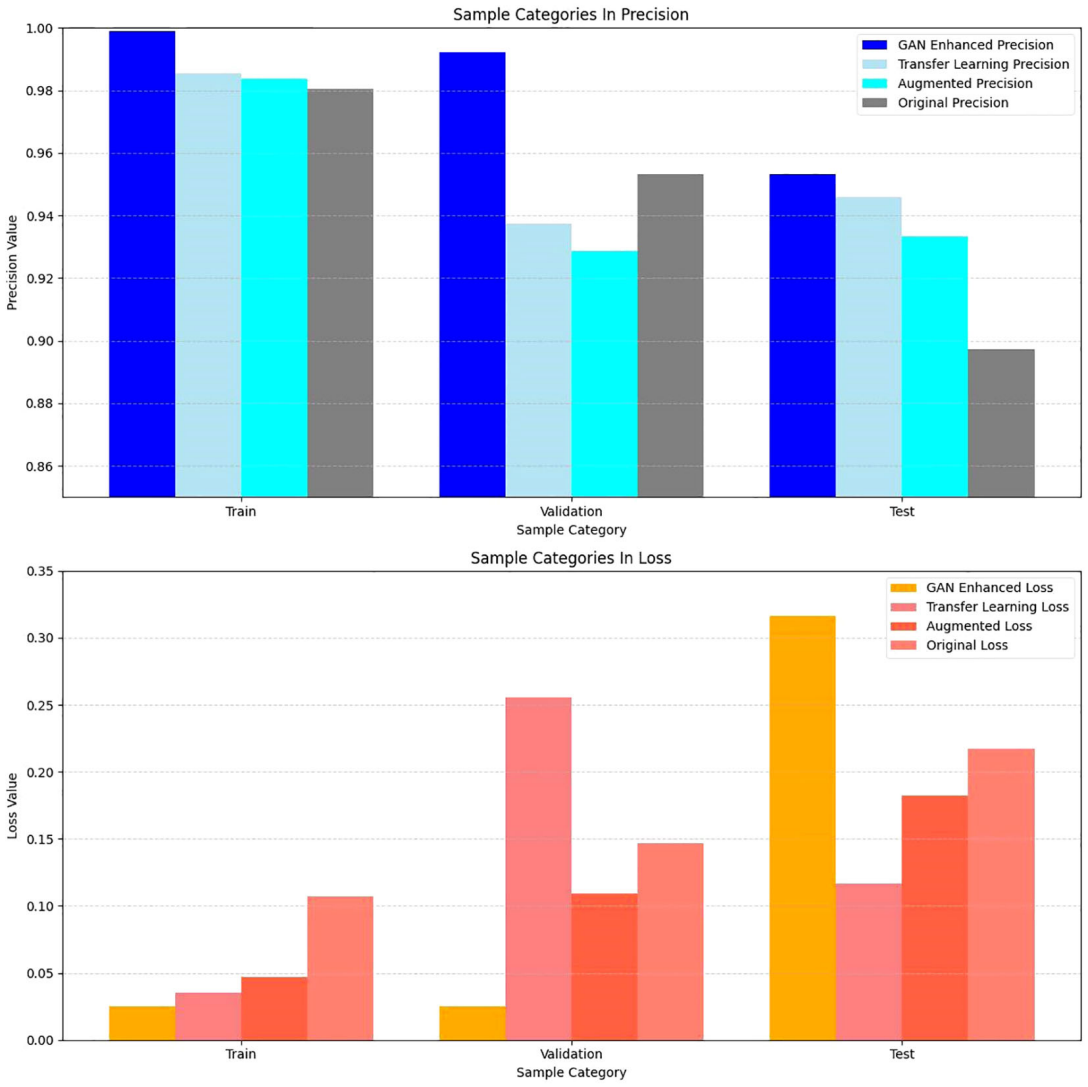
Comparison of Accuracy and Loss Values of Different Models on Different Datasets.

The data presented in the table are represented by the bar chart in **Figure 9**.

## 4 The outcome of the discussion

### 4.1 The impact of data augmentation techniques on the outcomes of training

Data augmentation is a commonly used technique that can increase the number of training datasets by transforming and expanding the original data. Data augmentation helps models learn more robust and generalized feature representations, thereby alleviating the problem of overfitting. Data augmentation can simulate diversity in the real world and improve the performance of a model in various scenarios. However, inappropriate or excessive data augmentation operations may introduce noise or unnecessary changes, leading to a decrease in the model performance.

In this study, the data enhancement technique mainly manipulates the experimental data such as rotation, noise, and changing exposure values. It significantly expands the training data set in a shorter time, and compared with the original data set on the convolutional network test, the use of data-enhanced samples really improves the robustness of the test model, effectively reduces the model’s under-training, and the model is more robust, which reduces the dependence on the training samples.

However, data augmentation shows some shortcomings for this small sample size. The performance of data augmentation in generating samples on this training set was not sufficient for the model to learn the complexity of the task adequately, and data augmentation introduced unwanted variations compared to other methods of expanding samples, and in some of these variations (brightness changes, noise additions) data augmentation resulted in some distortion of the images (darker images became darker, brighter images became brighter), which impacted the model’s ability to understand the real data and generalization ability. The data enhancement cannot completely replace the real data, because the enhanced data is based on the conversion of existing data and cannot completely replace the diversity and complexity of real data. Data augmentation also introduces some unwanted bias, and in the initial experiments, the bias in the augmentation settings resulted in very low performance of the test model on the sample set, which indicates a preference or over-augmentation of certain categories or features. This may lead to a decrease in model performance in some cases.

### 4.2 The impact of transfer learning techniques on training outcomes

By training models in a source domain, their knowledge can be transferred to a target domain, reducing the need for extensive labeled data. Transfer learning effectively uses existing data and models to achieve a better performance in the target task. However, the success of transfer learning is closely related to the similarity between source and target domains. If significant differences exist between the two domains, the effectiveness of transfer learning may be limited.

The InceptionV3 network used in this study was pre-trained on a large ImageNet dataset, which contains plant-specific data. Therefore, it is suitable for transfer learning of cotton wilt. Migration learning uses the knowledge of the original domain to help train small samples, and its does not require time for data manipulation for data augmentation, thus reducing the training time. In addition, migration learning introduces additional training samples that increase the amount of training data and help the model better learn the features and patterns of the target task.

However, for this training, there are still some differences between the original dataset and the target dataset, and in the convolutional network used for testing, this small difference will affect the training effect more. Although migration learning can use the knowledge and data of the source domain to increase the number of training samples, in the case of this experiment, the data of the source domain itself is limited, so the effect of migration learning is more limited.

### 4.3 The impact of generative adversarial network technology on training outcomes

Known as a generative adversarial network (GANs), the framework comprises a generator and a discriminator, engaging in a game-like training process where the generator aims to produce realistic data, while the discriminator tries to differentiate between real and generated data. GAN networks have demonstrated effective performance in tasks such as image synthesis and style transfer, generating images that exhibit realism and diversity. However, the training process of GAN is unstable, often leading to issues such as mode collapse and convergence.

In this study, a GAN structure with three distinct architectures was used. Through the continuous adjustment of the hyperparameters, a network structure that generated more realistic and clear samples was ultimately selected.

In the final model, the GAN network performs effective data augmentation and sample expansion, and it generates realistic synthetic data, which is particularly beneficial for training with limited samples. By generating additional samples, especially in the absence of rich data in the target domain, the GAN network can expand the training data set, thereby increasing the diversity and number of training samples. The GAN network, in this experiment, although caused overfitting of the convolutional network used for testing (so much realistic generated data should be tested with a more complex network, and in order to ensure the consistency of the experimental variables, a more complex network was not used for the three samples to ensure the consistency of the experimental variables), but its performance on the test set is also significantly due to the other two data expansion methods. The data generated by GAN can simulate the distribution characteristics of real data and help the model to better understand and adapt to the real data of the target task. As a result, the robustness and performance of the model are improved when dealing with real data.

However, the GAN network shows some shortcomings in this experiment, which are due to the characteristics of the GAN network. First of all, GAN network has the characteristic of training instability, due to the generator and discriminator against each other, its parameter transmission is unstable, so it is easy to have the phenomena such as gradient explosion and pattern collapse during the training process, so the GAN network needs more debugging and optimization to ensure the stability of the GAN and the quality of the generated data. At the same time, GAN networks require high computational resources, and training with a small sample size requires striking a balance between limited computational resources and training effects. Finally, in terms of generated data, although GAN networks can generate real data, there is still randomness and uncertainty in the generated samples, which need to be checked and used.

## 5 Conclusion

Using the power of Generative Adversarial Networks (GANs) can significantly enhance the precision of training in small-scale learning. GANs achieve this by generating aesthetically persuasive synthetic data, thereby expanding the training dataset and increasing its diversity. This data augmentation assists in alleviating the overfitting tendencies inherent in small-scale training, ultimately enhancing the generalizability of the model. The process of training a GAN aids the model in acquiring more refined and superior feature representations, subsequently bolstering the training accuracy.

By using the data generated by GANs, the model can more effectively adapt to the distribution and characteristics of real-world data. The versatility and authenticity of the generated data contribute to the model’s improved comprehension of the target task, along with augmented performance on unfamiliar data instances. Despite the potential challenges associated with the stability and computational resource requirements during GAN training, the advantages of leveraging GAN-generated data in small-scale learning far outweigh any obstacles encountered.

Data augmentation and feature learning based on GANs substantially enhanced the training accuracy of small-scale learning. By augmenting the dataset, mitigating overfitting, and simulating the distribution of real-world data, this approach provided a model with an enhanced training foundation. Hence, in small-scale learning tasks, the use of Generative Adversarial Networks is a valid and effective strategy that enables improved model performance and yields superior training outcomes.

## Data availability statement

The raw data supporting the conclusions of this article will be made available by the authors, without undue reservation.

## Author contributions

ZHZ: Conceptualization, Data curation, Formal analysis, Investigation, Methodology, Validation, Visualization, Writing – original draft, Writing – review & editing. LM: Resources, Writing – review & editing. CW: Writing – review & editing. MY: Resources, Writing – review & editing. SQ: Investigation, Methodology, Writing – review & editing. XL: Writing – review & editing. ZZ: Writing – review & editing.
