# Supplementary material for: Cotton Fusarium wilt diagnosis based on generative adversarial networks in small samples
Source: Front Plant Sci. 2023 Dec 11;14:1290774. doi: 10.3389/fpls.2023.1290774 (PMC10754962; doi:10.3389/fpls.2023.1290774)
Supplement: Supplementary file 1 [file DataSheet_1.pdf]

Supplementary Material

1 Supplementary Data

Supplementary Table 1 Hyperparameters of a convolutional neural network

Super parameter	Value
Learn Rate	0.01
Batch	32
Epoch	20
L2	0.001
Dropout	0.5
Optimizing algorithm	Adam

Supplementary Table 2 Hyperparameters for InceptionV3

Super parameter	Value
Weights	ImageNet
Include Top	Flase
Epoch	20
Dense	1024
Activation	relu
Optimizing algorithm	Adam

Supplementary Table 3 Typical hyperparameters of a generator

Super parameter	Value
Learn Rate	0.0004
Batch	16
L2	0.001
LeakyReLU(1)	0.2
LeakyReLU(2)	0.3
ClipValue	1.0
Decay	1e-8
Optimizing algorithm	Adam

Supplementary Table 4 Hyperparameters of a discriminator

Super parameter	Value
Learn Rate	0.0001
Batch	16
LeakyReLU	0.2
Dropout	0.5
Decay	1e-8
Optimizing algorithm	Adam

2 Supplementary Figures and Tables

2.1 Supplementary Figures

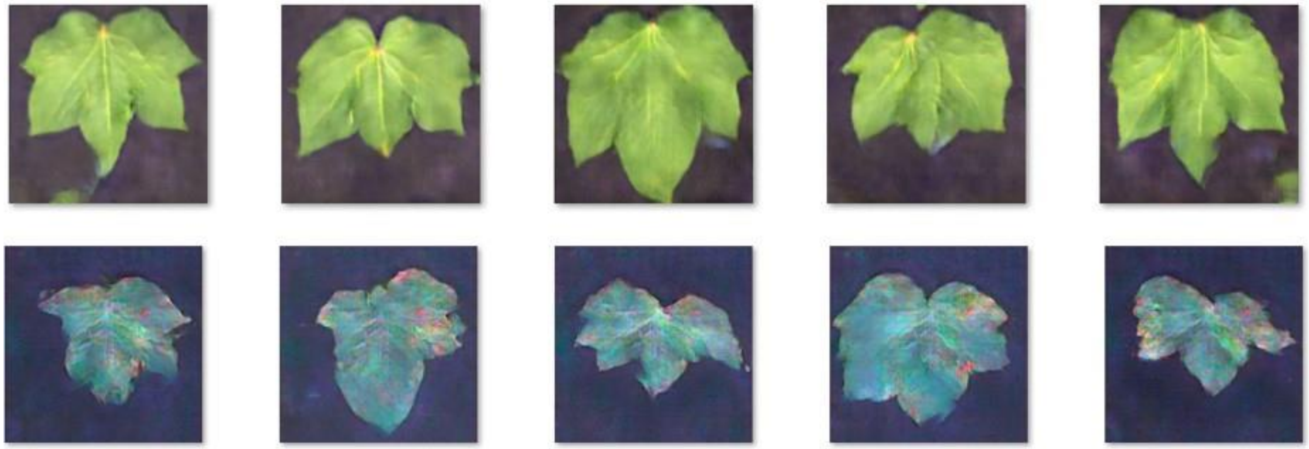

Supplementary Figure 1. Example of a part of the image generated by the adapted DCGAN network

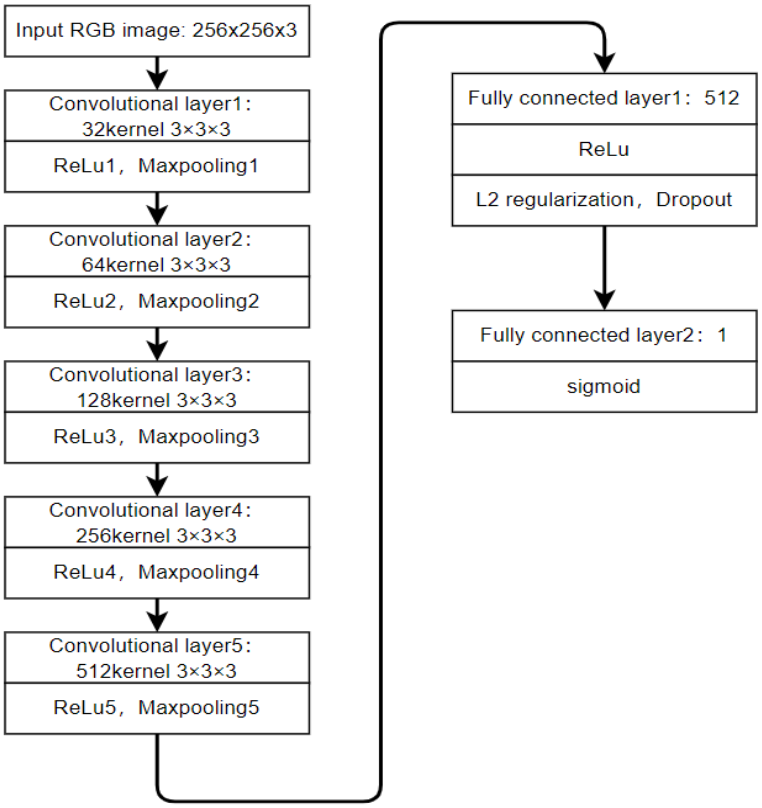

Supplementary Figure 2. Schematic diagram of CNN network structure

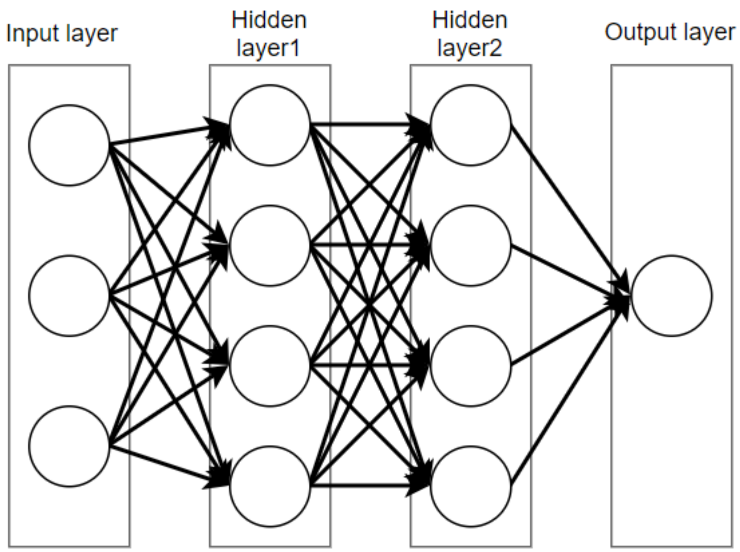

Supplementary Figure 3. Fully Connected Network (FC) and Convolutional Network

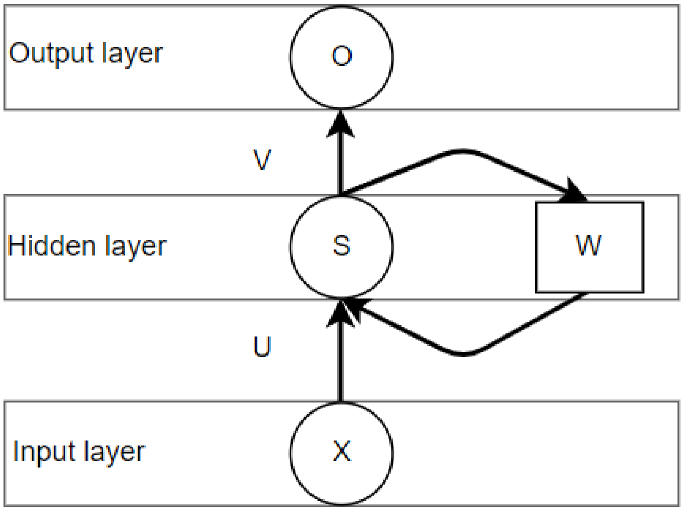

Supplementary Figure 4. Recurrent Neural Network (RNN)

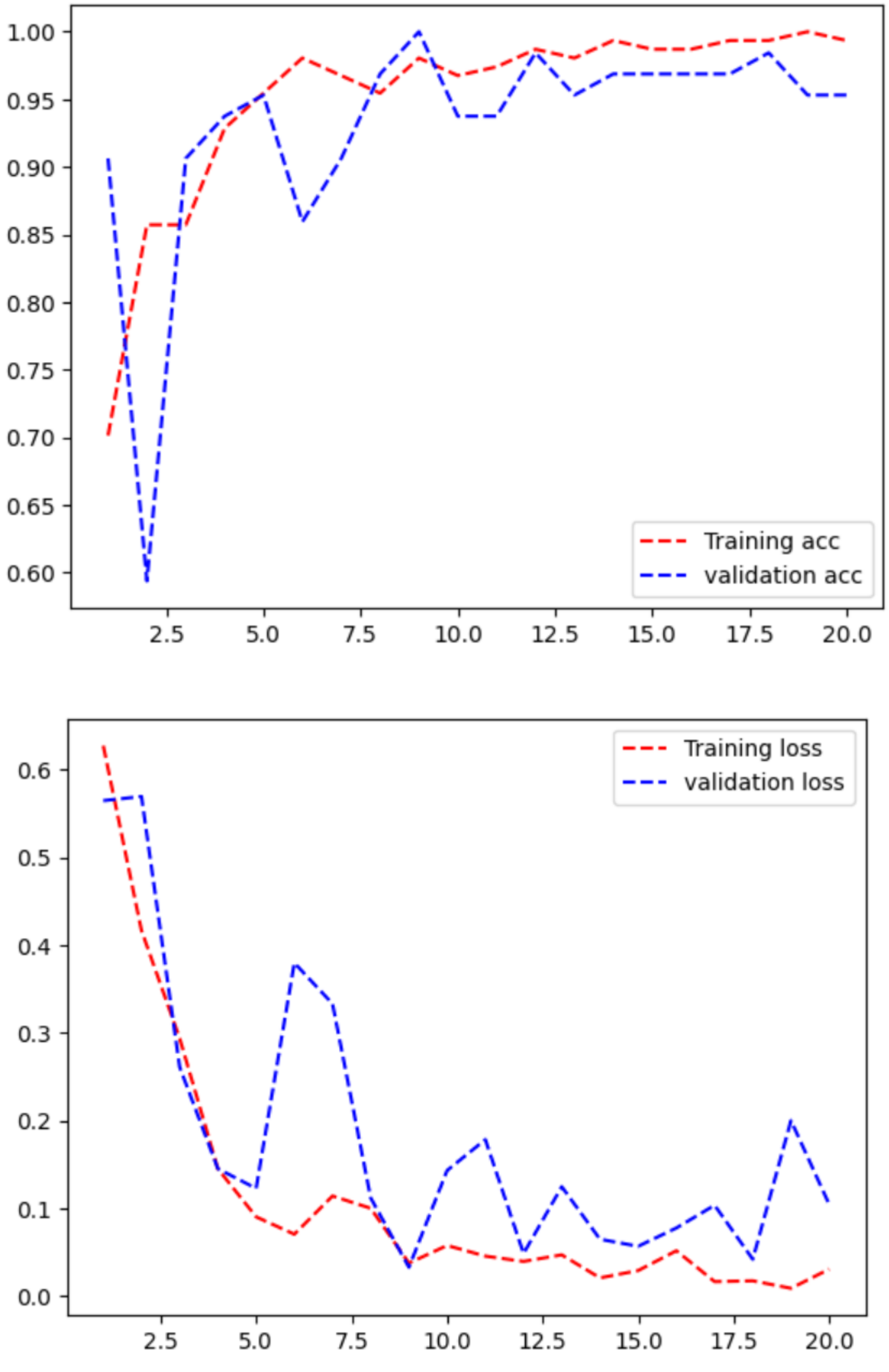

Supplementary Figure 5. Results of the original data on the VGG19 pre-trained network

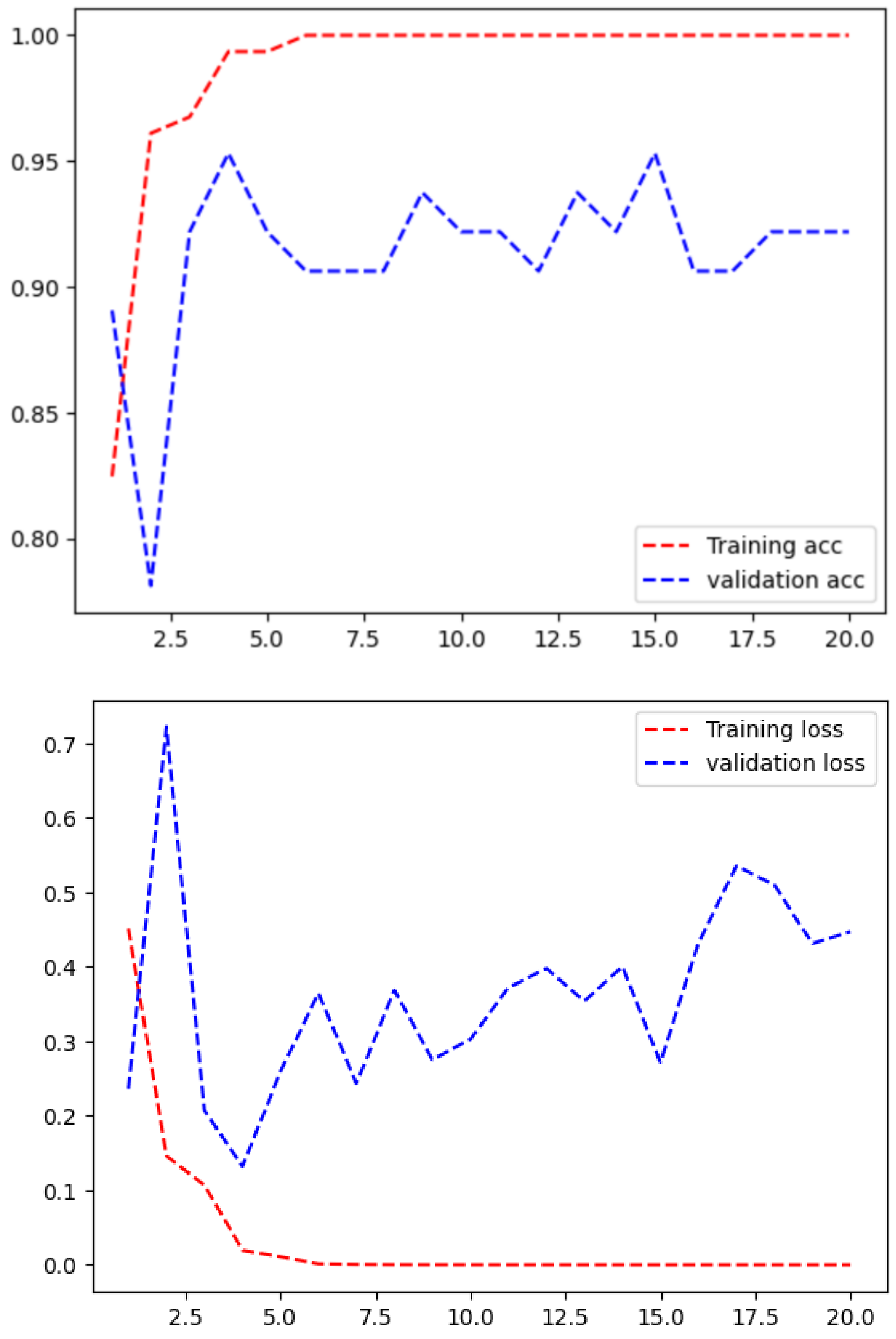

Supplementary Figure 6. Results of the original data on the ResNet50V2 pre-trained network

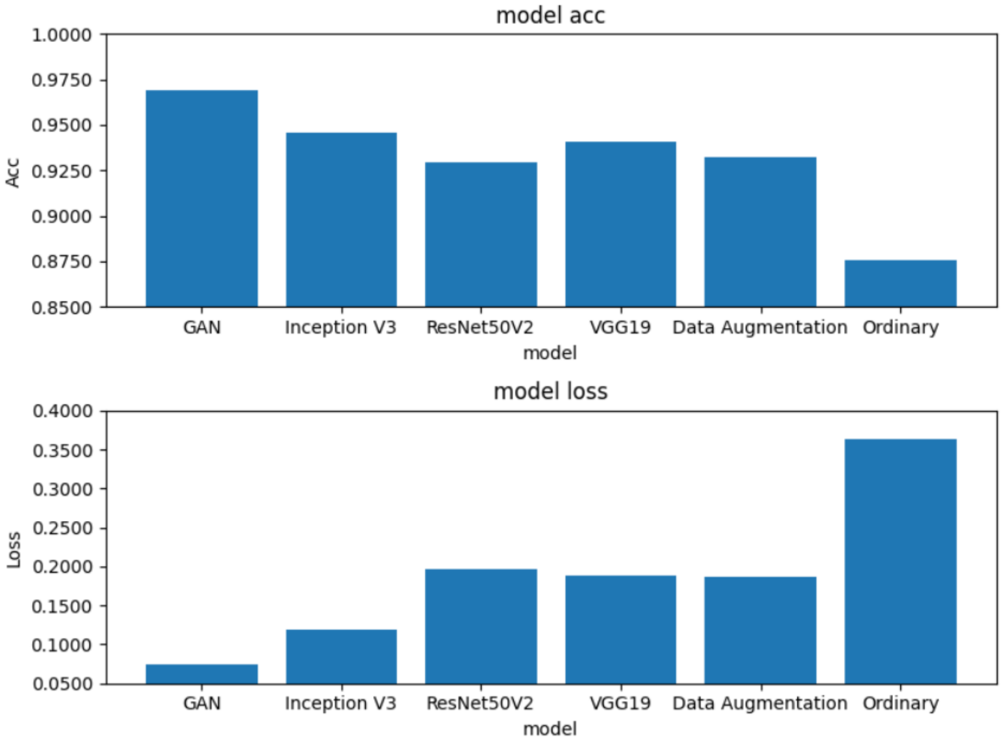

Supplementary Figure 7. Comparison of individual networks on the test set
